# Changes in the intestinal microbiota of superobese patients after bariatric surgery

**DOI:** 10.6061/clinics/2019/e1198

**Published:** 2019-10-23

**Authors:** Denis Pajecki, Lea Campos de Oliveira, Ester Cerdeira Sabino, Marcela de Souza-Basqueira, Anna Carolina Batista Dantas, Gabriel Cairo Nunes, Roberto de Cleva, Marco Aurélio Santo

**Affiliations:** I Unidade de Cirurgia Bariatrica e Metabolica, Departamento de Gastroenterologia, Hospital das Clinicas HCFMUSP, Faculdade de Medicina, Universidade de Sao Paulo, Sao Paulo, SP, BR.; II Laboratorio de Medicina Laboratorial (LIM03), Hospital das Clinicas HCFMUSP, Faculdade de Medicina, Universidade de Sao Paulo, Sao Paulo, SP, BR.; III Departamento de Molestias Infecciosas, Faculdade de Medicina FMUSP, Universidade de Sao Paulo, Sao Paulo, SP, BR.; IV Instituto de Medicina Tropical de Sao Paulo, Sao Paulo, SP, BR.; V Laboratorio de Parasitologia (LIM46), Hospital das Clinicas HCFMUSP, Faculdade de Medicina, Universidade de Sao Paulo, Sao Paulo, SP, BR.

**Keywords:** Gut Microbiota, Fecal, Obesity, Bariatric Surgery, Gastric Bypass

## Abstract

**OBJECTIVES:**

The gut microbiota is associated with obesity and weight loss after bariatric surgery and has been related to its changing pattern. Exactly how the bacterial population affects weight loss and the results of surgery remain controversial. This study aimed to evaluate the intestinal microbiota of superobese patients before and after gastric bypass surgery (RYGB).

**METHOD:**

DNA fragments for the microbiota obtained from stool samples collected from nine superobese patients before and after bariatric surgery were sequenced using Ion Torrent.

**RESULTS:**

We observed that with a mean follow-up of 15 months, patients achieved 55.9% excess weight loss (EWL). A significant population reduction in the Proteobacteria phylum (11 to 2%, *p*=0.0025) was observed after surgery, while no difference was seen in Firmicutes and Bacteroidetes. Further analyses performed with two specific individuals with divergent clinical outcomes showed a change in the pattern between them, with a significant increase in Firmicutes and a decrease in Bacteroidetes in the patient with less weight loss (%EWL 50.79 *vs*. 61.85).

**CONCLUSIONS:**

RYGB affects the microbiota of superobese patients, with a significant reduction in Proteobacteria in patients with different weight loss, showing that different bacteria may contribute to the process.

## INTRODUCTION

Obesity is a chronic disease that affects more than 600 million people worldwide (1). The pathophysiology is considered multifactorial, with the gut microbiota influencing the energy balance, the inflammatory state, the intestinal barrier and the regulation of food intake, leading to an increase in body weight (2).

An experimental study showed a higher proportion of the Firmicutes phylum in genetically obese mice than in eutrophic controls, with a change in the ratio of Firmicutes/Bacteroidetes after weight loss (3). In humans, a lower proportion of Bacteroidetes has also been observed in obese patients than eutrophic controls, in addition to changes in the amount of Actinobacteria (4). A higher proportion of Firmicutes is associated with greater energy absorption, while more Bacteroidetes is associated with a decrease (5). Nevertheless, the participation of genera and species also seems important since the distribution of phyla between lean and obese individuals shows no difference in some studies (6).

Bariatric surgery is considered the gold standard treatment for morbid obesity. Despite the rise of sleeve gastrectomy, Roux-en-Y Gastric Bypass (RYGB) is still the most accomplished weight loss procedure in Brazil (7). Patients submitted to this technique 3 to 12 months postoperatively present a reduction in Firmicutes and Bacteroidetes and an increase in Gammaproteobacteria (8). Considering genera and species, an increase in *E. coli* and reduction in *Bifidobacteria* and *Lactobacillus* has been observed (9). However, there is an uncertain significance of these findings, and recent conflicting data show an increase in Firmicutes and reduction in Bacteroidetes in some individuals, which is associated with variations in diabetes control in the postoperative period (10).

The influence of factors such as diet, environment and medication use is certain in long-term follow-up after bariatric surgery (11), but it is not known how the intestinal microbiota influences this process. Satisfactory sustained weight loss is commonly achieved after bariatric surgery, with 65% excess weight loss (%EWL) after RYGB (12). Nevertheless, weight regain and recurrence of obesity is a major concern in long-term follow-up, with multifactorial causes (13,14). It is estimated that up to 20% of patients will present with treatment failure, particularly among superobese patients (BMI above 50 kg/m^2^) (15).

Consequently, in this study, we evaluated the intestinal microbiota of superobese patients before and after the RYGB technique.

## MATERIALS AND METHODS

**Ethics approval and consent to participate:** All procedures involving human subjects were approved by the Internal Review Board of the University of São Paulo (“Comissão de Ética para Análise de Projetos de Pesquisa” - CAPPesq number 399.864, 09/19/2013). Participants provided written consent, which was securely stored in our laboratory, according to Brazilian research policy.

### Study population

The study enrolled nine superobese patients submitted to bariatric surgery from 2014 to 2015 at Hospital das Clínicas, University of São Paulo, Medical School, at São Paulo, Brazil. Inclusion criteria were BMI≥50 kg/m^2^, and exclusion criteria were the use of antibiotics or acute diarrhea three months prior to surgery, chronic diarrhea, inflammatory bowel disease and previous gastrointestinal surgery (including revisional bariatric surgery).

Fecal samples for microbiota study were collected before and 12 to 24 months after surgery. The surgical technique was RYGB with both alimentary and biliopancreatic limbs of 100 cm each. Postoperative follow-up examinations were performed routinely at 1, 3, 6, 12, 18 and 24 months. This study was approved by the ethics committee (CAPPesq number 399.864).

### Collection and storage of stool samples

A Fisher Fecal Commode Collection Kit was used to collect the stool samples, and they were placed at -80°C up to 1 hour after collection and maintained there until DNA extraction.

### DNA extraction

Fecal DNA extraction was performed using a Power Soil DNA Isolation Kit^®^ (Mobio Laboratories, Carlsbad, CA), with modifications (16). Briefly, the sample tubes were heated for 10 minutes at 65°C and a further 10 minutes at 95°C and then centrifuged for 2 minutes after the addition of C3 solution. All other steps were performed according to the manufacturer's instructions.

### Library preparation and 16S sequencing

The V4 variable region of the 16S rRNA gene was amplified using the primers 515F (5′-GTGCCAGCMGCCGCGGTAA-3′) and 806R (5′-GGACTACHVGGGTWTCTAAT-3′) (17). These primers were designed to include the adaptor sequences used in the Ion Torrent sequencing library preparation protocol, containing the barcode sequence on the forward primer. Samples were normalized to 12.5 ng/μl DNA material per library, and the amplification was performed using a Veriti 96 well PCR (Applied Biosystems) followed by AMPure XP bead cleanup (Beckman Coulter). The PCR conditions used were 94°C for 3 minutes, followed by 40 cycles of denaturation at 94°C for 30 seconds, annealing at 58°C for 30 seconds and extension at 68°C for 1 minute. PCR products were analyzed by 1.5% agarose gel electrophoresis. PCR emulsion was carried out using an Ion PGM™ Template OT2 400 Kit in accordance with the manufacturer’s instructions. Sequencing was carried using an Ion 318^TM^ chip kit v2, with 16 libraries per chip, using an Ion PGM™ Sequencing 400 Kit, on an Ion Torrent^TM^ Personal Genome Machine (ThermoFisher, USA). All the samples were sequenced once.

### Data analysis

The obtained sequences were processed using the Ion Torrent server v5.0.4. Low quality and polyclonal sequences were excluded by filtering. Reads maintained were grouped into operational taxonomic units (OTUs) based on 97% identity using UCLUST UPARSE v7 (18). The representative sequences were then classified by taxonomy using the Greengenes database v13.8 (19) as a reference on the QIIME (Quantitative Insights Into Microbial Ecology) software package v1.8 (20).

### Statistical analysis

The species richness/diversity were assessed by pairwise comparisons for alpha diversity by OTUs, Shannon diversity index, Chao1 richness estimate, and Simpson diversity index. To determine the effect of surgery on the shared diversity between samples, beta diversity ratings were calculated based on weighted and unweighted UniFrac distance matrices, comparing samples pre- and post-bariatric surgery.

To determine differences in the microbiota before and after bariatric surgery, the nonparametric Kruskal-Wallis test was applied. To compare the percentage of bacteria present before and after the surgery in two patients, the Chi-squared test was used. All analyses were performed using GraphPad Prism 6^TM^ statistical software. A *p*-value of <0.05 after Bonferroni correction was considered statistically significant.

## RESULTS

The clinical and epidemiological data from the patients included in the study are presented in Table 1. Most patients were female and Caucasian, with a mean age of 41.9 years and a preoperative BMI of 56.47 kg/m^2^. In a mean follow-up of 15 months, they achieved an EWL of 55.9%.

**Figure 1:**
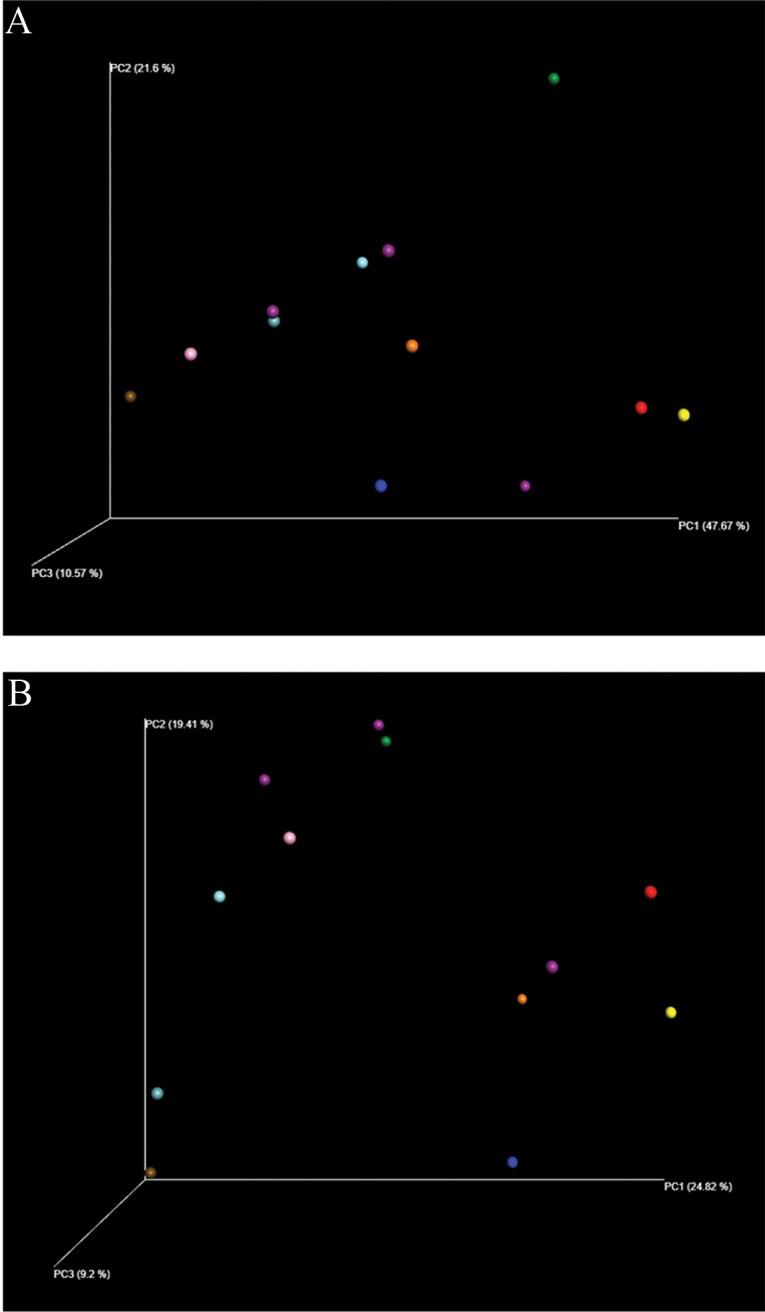
PCoA analysis based on UniFrac distance matrices comparing the abundance of intestinal bacteria before and after surgery. A: unweighted; B: weighted.

The analyses were performed by grouping all patient data before and after surgery. The samples were grouped with greater proximity related to the surgical status and compared by the diversity analysis of the samples via the UniFrac method (Figure 1).

**Figure 2:**
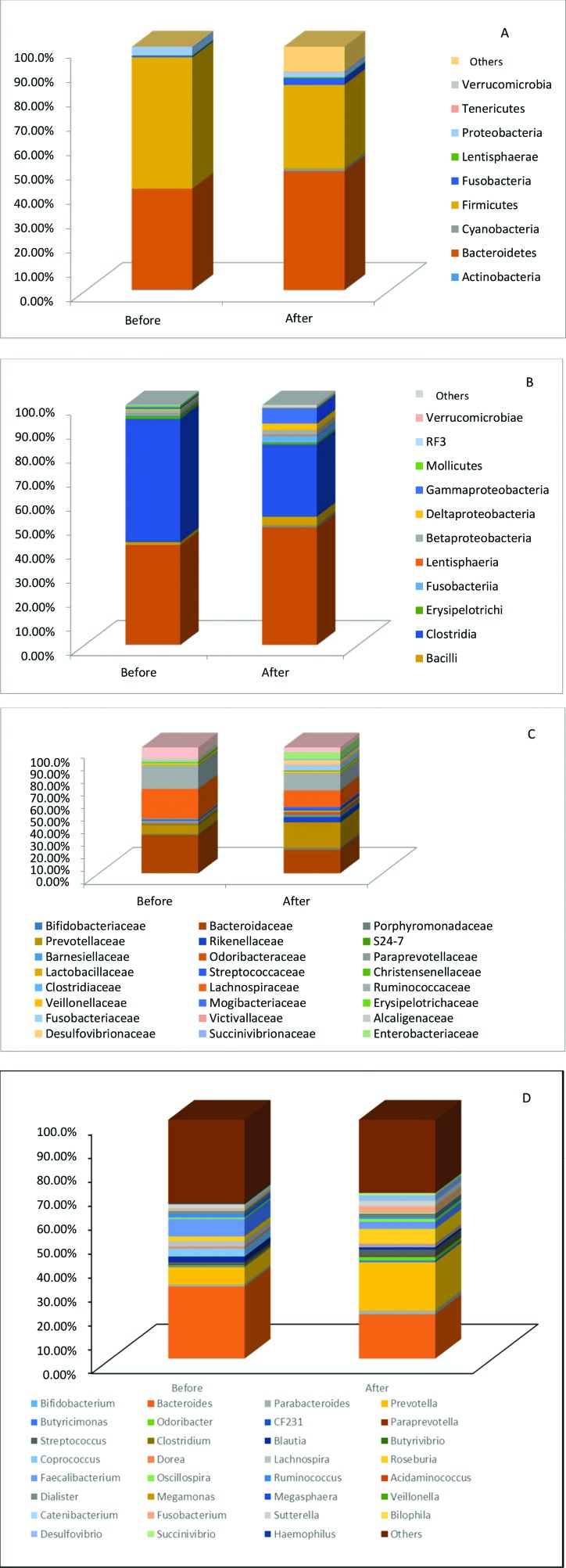
Relative abundance at the phyla, class, family and genus levels of fecal samples, comparing patients before and after surgery. A – Phyla; B – Class; C – Family; D – Genus.

A significant reduction in the Proteobacteria phylum (11% to 2%, *p*=0.0025) was observed after surgery, but no significant difference was seen in Firmicutes and Bacteroidetes. Differences at the family level for Rikenellaceae, Enterobacteriaceae, Sucinivibroniaceae and Odoribacteriaceae and at the genus level for *Roseburia* were not maintained after Bonferroni correction (Figure 2).

**Figure 3:**
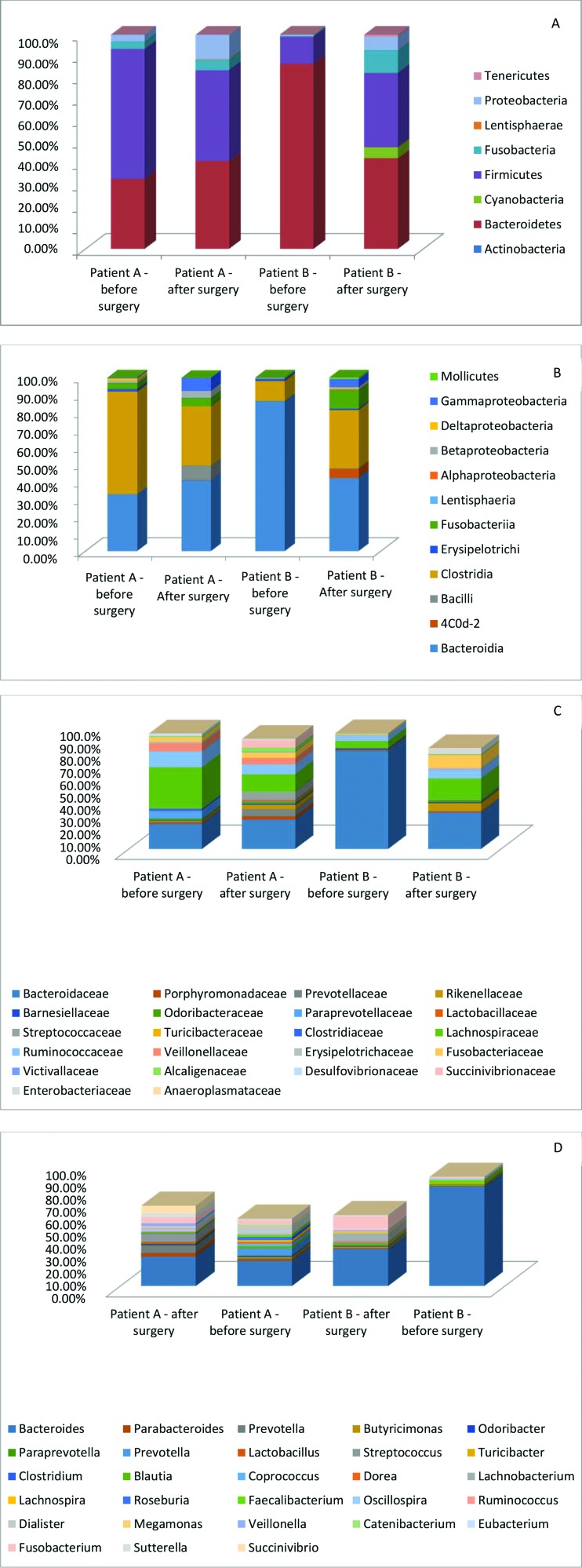
Relative abundance at the phyla, class, family and genus levels of fecal samples, comparing patients 1 and 3 prior to and post surgery. A – Phyla; B – Class; C – Family; D – Genus.

Further analyses were performed with two individuals, comparing the data before and after surgery. These patients showed opposite results of weight loss. Patient A had a 61.85% EWL, and patient B had a 50.79% EWL. Considering the bacterial abundance at the phylum level, the abundance of Bacteroidetes and Firmicutes was different pre- and postoperatively. Bacteroidetes was significantly reduced in patient B after surgery (86 to 42%, *p*<0.0001). Conversely, Firmicutes was diminished in patient A (61 to 42%, *p*=0.0107) and increased in patient B (13 to 35%, *p*=0.0004). At the class level, Bacteroidia and Clostridia showed trends similar to those of their phyla, Bacteroidetes and Firmicutes, respectively. The Clostridia class was significantly reduced after surgery in patient A (59% to 34%, *p*=0.0006) and increased in patient B (11% to 33%, *p*=0.0006). At the family level, Bacteroidaceae and Lachnospiraceae maintained trends similar to those of their corresponding class and phyla, Bacteroidia/Bacteroidetes and Clostridia/Firmicutes, respectively. Furthermore, Bacteroidaceae was significantly reduced in patient B (85% to 31%, *p*<0.0001), and Lachnospiraceae was reduced in patient A (36% to 15%, *p*=0.0011) and increased in patient B (6% to 18%, *p*=0.0153) (Figure 3).

## DISCUSSION

This longitudinal study utilizing high-throughput Ion Torrent sequencing in superobese patients before and after bariatric surgery showed a statistically significant reduction in the Proteobacteria phylum after surgery and no significant changes in Firmicutes and Bacteroidetes. These findings are in contrast with recent studies relating Proteobacteria to inflammation, dysbiosis and extraintestinal diseases (21).

Changes in the microbiota following bariatric surgery are related to surgical technique, and a meta-analysis of six studies demonstrated that RYGB caused an increase in Proteobacteria and a decrease in Firmicutes (22). Conflicting data regarding the Firmicutes and Bacteroides phyla are related to obesity and bariatric surgery (3). These converse findings are often related to the diet (23,24) and changes in acid exposure due to surgical technique and routine use of proton pump inhibitor (PPI) drugs (8,9). In our study, no patient used long-term PPI treatment.

Two patients with divergent weight loss outcomes individually analyzed showed inverse alterations of Firmicutes, Clostridia and Lachnospiraceae. Additionally, the patient with the worst weight loss (%EWL of 50.79%) had a significant decrease in Bacteroidetes and increase in Firmicutes. Although this similar order is seen in obese patients in clinical studies, the differences at the phylum level between lean and obese individuals have shown conflicting outcomes (5,6).

Some limitations in our study were observed, mostly related to the small sample size. Likewise, our findings could be affected by uncontrolled factors, such as postoperative diet. The surgical technique chosen can also affect the study outcome, but RYGB is the most frequent technique in microbiota studies (22).

The bacterial region and the methods used to detect them are very important to assessing the microbiota, and they can influence the results. The V4 region of the 16S bacterial RNA gene, which contains both conserved and variable regions, is commonly sequenced to identify bacterial species and was used in this study. Prior studies present different methods ranging from simple amplification of the 16S RNA gene by PCR to next-generation sequencing. Semiconductor sequencing was used in the present study. This methodology has limitations, such as pairing errors and homopolymer limited detection, which must be taken into account, according to the type of study (25). Different approaches have been used in the Ion Torrent platform analyses to minimize these errors (26). Even using the same data, the methodology chosen for analysis may result in different findings (27).

To what extent the changes in the microbiota of this group of patients may influence weight loss or regain is still uncertain. Further knowledge of these modifications, with the identification of species that may have a more positive effect in this process, could contribute to the development of microbiota modulation therapies with prebiotics, probiotics or even fecal transplantation in operated patients.

Our findings support previous literature outcomes of changes in the microbiota after surgery, with a significant reduction in Proteobacteria associated with mostly inflammation and extraintestinal diseases. Additionally, when comparing data from patients with different clinical outcomes, we observed that Firmicutes and Bacteroidetes may not be responsible for the observed phenotype, and other bacteria, even in lower proportions, may not be disregarded.

## AUTHOR CONTRIBUTIONS

Pajecki D, Sabino EC and Santo MA conceived the study. Oliveira LC, Souza-Basqueira M and Dantas ACB were responsible for the formal analysis. Sabino EC was responsible for funding acquisition. Pajecki D, Oliveira LC, Souza-Basqueira M, Dantas ACB, Nunes GC and De Cleva R were responsible for the investigation. Oliveira LC, Souza-Basqueira M, Dantas ACB and Nunes GC were responsible for the methodology. De Cleva R and Santo MA supervised the study. Pajecki D, Sabino EC, De Cleva R and Santo MA were responsible for the visualization. Oliveira LC was responsible for the manuscript original drafting. All of the authors provided assistance in manuscript writing, review, editing, and read and approved the final version of the manuscript.

## Figures and Tables

**Table 1:** Demographic and weight data from the patients submitted to bariatric surgery.

	N=9
Female gender, n (%)	6 (66.7%)
Age, years (min-max)	41.9 (16 – 59)
Caucasian ethnicity, n (%)	8 (88.8%)
Preoperative BMI, kg/m^2^	56.47 (50.69 – 62.87)
Postoperative BMI, kg/m^2^	38.74 (36.73 – 39.75)
%EWL	55.89 (50.79 – 61.85)
